# Exploring the status of online social support for older adults with cancer: a scoping review protocol

**DOI:** 10.1136/bmjopen-2024-087251

**Published:** 2024-12-26

**Authors:** Fei Liu, Sophie Pilleron, India Pinker

**Affiliations:** 1 Ageing, Cancer and Disparities Research Unit, Department of Precision Health, Luxembourg Institute of Health, Strassen, Luxembourg; 2 Faculty of Humanities, Education and Social Sciences, University of Luxembourg, Esch-sur-Alzette, Luxembourg

**Keywords:** Aged, Internet, ONCOLOGY, Quality of Life, Social Support

## Abstract

**Introduction:**

The estimated number of new cases among older adults with cancer has been increasing. Considering the decrease in social networks as adults age, their need for social support is often unmet. Notably, an increasing number of older adults with cancer have access to social support through online technologies, especially since the COVID-19 pandemic, which heightened the need for online social support. Little is known, however, about the extent to which online social support for older people with cancer has developed. This scoping review aims to explore the developments in online social support for older adults with cancer.

**Methods and analysis:**

We will search PubMed, Elsevier Embase (including Medline) and EBSCO CINAHL Complete to identify eligible studies based on predefined criteria. Screening of articles and data extraction will be carried out independently by two reviewers, with conflicts resolved by a third reviewer. This scoping review will be based on the Arksey and O’Malley methodological framework with the extension proposed by Levac and colleagues. The review findings will be presented in a narrative analysis using tables. This protocol is registered on Open Science Framework at https://doi.org/10.17605/OSF.IO/Z9XJ7.

**Ethics and dissemination:**

No ethical approval is needed. The findings will be published in a peer-reviewed journal and presented at conferences.

**STRENGTHS AND LIMITATIONS OF THIS STUDY**

- The search strategy was conducted in collaboration with a research librarian.
- There are no restrictions on study period, geography or languages to create a more comprehensive search.
- This is the first scoping review study on the use of online social support for older adults with cancer.
- A limitation of this scoping review is the exclusion of grey literature, which could omit relevant studies that are not published in peer-reviewed journals.
- The lack of a quality assessment, as stipulated by the chosen methodology, may introduce biases due to variations in the quality of the included articles, potentially limiting the generalisability of the findings.

## Introduction

The global population is ageing, which has led to a rising incidence of cancer among adults aged 65 years and older worldwide.^1^ Based on the latest Global Cancer Observatory (GLOBOCAN) estimates, the annual number of new cancer diagnoses among older adults is predicted to increase by 112% from 9.6 million in 2022 to 20.4 million in 2050.^2^

Older adults with cancer are a heterogeneous group, often burdened with pre-existing health conditions (eg, chronic diseases, impaired physical and cognitive function).^3 4^ Studies have found a widening gap in needs and survival probabilities between younger and older adults with cancer.^5^ Older adults with cancer often have complex needs, both physically and psychologically, leading to challenges in receiving adequate support and care.^3 6^ In addition to face-to-face support that addresses their complex needs, online social support can also provide a sense of connection and community, regardless of geographical constraints.^7^ As such, vulnerable and frail older adults with cancer, in particular those who may not be able to travel easily, may benefit from social support provided through these online technologies.

As adults age, their social support networks can recede, a phenomenon that can exacerbate feelings of loneliness and heighten the impact of cancer-related stressors.^8^ Studies have suggested that robust social support mechanisms not only reduce psychological stress but can also inhibit tumour progression.^8^,^10^ Thus, given the increased need for yet diminished access to social networks, particularly among those experiencing loneliness, isolation or a lack of spousal support, research increasingly highlights unmet needs for social support among older adults with cancer.^4^ Recognising the multifaceted nature of cancer care, it becomes evident that medical interventions alone are insufficient in addressing the holistic needs of patients, older patients need further psychosocial support throughout the care pathway.^11^

Social support, as defined by the National Cancer Institute, is ‘a network of family, friends, neighbours and community members that is available in times of need to give psychological, physical and financial help’.^12^ It has been classified into four dimensions: esteem/emotional support, social companionship, informational support and instrumental support.^13^ Studies demonstrate that social support has a direct impact on cancer patients’ emotional health, physical health and quality of life.^14 15^ Notably, Mazzella *et al* have shown an association between low social support and a higher mortality risk among older adults with comorbidity, and Kroenke *et al* observed similar findings in women with breast cancer.^16 17^ Conversely, higher levels of social support have been linked to improved mental health and enhanced quality of life among older adults.^18^

Despite lower rates of internet adoption among older adults compared with younger adults, recent research indicates that there has been a significant uptake of digital technologies among older adults in the past decades, particularly in the context of telehealth and online platforms.^19 20^ This trend of older adults adopting online tools has accelerated since the beginning of the COVID-19 pandemic.^19^,^21^

Online social activities enable older adults to connect and engage in digital interactions during the pandemic.^22^ Researchers have begun to investigate the feasibility of an online patient health community to support older women with breast cancer.^23^ Moreover, some studies revealed positive attitudes towards the adoption of digital health technologies among older adults with cancer, especially after exposure to telemedicine services during the COVID-19 pandemic.^24 25^ However, little is known about the extent to which online social support for older people with cancer has developed. This scoping review aims to provide an overview of the literature on online social support for older adults with cancer.

Specifically, we aim to:

- Describe the characteristics of the online social support resources and interventions for older adults with cancer.
- Describe the barriers and facilitators to online support resources and interventions for older adults with cancer.
- Identify gaps in the literature.

## Methods and analysis

This protocol is based on Preferred Reporting Items for Systematic Reviews and Meta-Analyses extension for Scoping Review (PRISMA-ScR) guidelines.^26^

### Design

A scoping review will be carried out based on the Arksey and O’Malley methodological framework with the extension proposed by Levac and colleagues.^27 28^ The reporting of the article searches will be based on PRISMA-ScR guidelines for scoping reviews.^26^ A preliminary search was conducted in February 2024 in PubMed, PROSPERO and Open Science Framework. No systematic reviews or scoping reviews on the topic are currently underway.

### Patient and public involvement

No patient is involved.

### Eligibility criteria

The eligibility criteria were framed in terms of Participants, Intervention, Comparator and Outcomes (PICO—see table 1).^29^

**Table 1:** Eligibility criteria based on the PICO framework

Element	Inclusion criteria	Exclusion criteria
Participants	Studies involving adults with cancer aged 65 years and above (the mean/median ≥65 years old; or at least one group of participants is 65 and above).No restriction on cancer type.No restriction on comorbidities.	Studies focusing on the younger adult cancer population (<60 years old).Studies not related to cancer.
Intervention or exposure of interest	Any interventions related to online social support including any digital technology and online forms (including apps, websites, etc).Studies examining the feasibility, effectiveness or acceptability of online social support for older adults with cancer.	Studies focusing only on non-digital forms of social support (eg, in-person support groups and social support not facilitated via internet use).Studies focusing on digital interactions with healthcare professionals (both formal and informal telehealth).
Comparator	Any comparators related to digital social support for older adults with cancer(eg, different age groups but one must include older adults >65 years old or different social support interventions but one must include online social support intervention).Comparisons are not mandatory for inclusion.	
Outcome	Any outcomes related to online social support for older adults with cancer.	
Study design	Any quantitative design.Any qualitative design.Mixed methods designs.	Case reports.Reviews (eg, literature, narrative, systematic).
Article type	Original peer-reviewed research articles.	Perspective or opinion pieces, book chapters, dissertations, editorials, protocols and conference abstracts.
Language*	No restriction	
Study period	No restriction	
Geography	No restriction	
Study period	No restriction	

* Authors’ institution hosts diverse language expertise to support this criteria.

### Information resources

We will search for articles from PubMed, Elsevier Embase (including Medline) and EBSCO CINAHL Complete, as advised by our in-house research librarian for optimum literature coverage across the institutionally available databases.

### Search

The search strategy was developed with the support of a health-focused research librarian (see table 2). The search was formulated in PubMed using a combination of interface command language and text keywords as well as Boolean operators and truncation. Then, the strategy will be translated into other databases, reflecting their command languages, when applicable. To ensure the most up-to-date literature is included, this search will be run once more before the final analysis to ensure we capture as much relevant literature as possible.

**Table 2:** Key concepts and preliminary search terms

Key concept	Search terms	MeSH terms
Online	online[TW] OR digital[TW] OR technology[TW] OR “e-health”[TW] OR “social media”[TW] OR Internet[TW] OR web[TW] OR forum[TW] OR “chat room”[TW] OR “chat group” OR “e-bulletin”[TW] OR “instant messaging”[TW] OR “mailing list”[TW] OR “virtual support”[TW] OR app[TW] OR apps[TW] OR Facebook[TW] OR Whatsapp[TW] OR zoom[TW] OR “Microsoft teams”[TW] OR Webex[TW] OR “virtual platform”[TW] OR Skype[TW] OR Facetime[TW] OR"video call*“[TW] OR “smart device”[TW]	
Social support	“Social Support”[MH] OR “Social Support”[TW] OR “social work”[MH] OR “social work”[TW] OR “social service”[TW] OR “public support”[TW] OR “social help”[TW] OR communit*[TW] OR “social connection”[TW] OR social network*[TW] OR “social engagement”[TW] OR “social context”[TW]OR"social relationship*“[TW] OR"social ties”[TW] OR"social contact*“[TW] OR “social integration”[TW] OR "social resource*“[TW] OR friendship*[TW] OR "emotional support”[TW] OR "tangible support”[TW] OR “informational support”[TW] OR “instrumental support”[TW] OR “companionship support”[TW] OR "perceived support”[TW] OR “received support”[TW]	Social supportSocial work
Older adults	Aged[MH] OR “Older adult*”[TW] OR “oldest old”[TW] OR “older patient*”[TW] OR “old age”[TW] OR aging[TW] OR ageing[TW] OR Elder*[TW] OR “Geriatrics”[MeSH] OR Geriatric*[TW] OR Geronto*[TW] OR Nonagenarian*[TW] OR Octogenarian*[TW] OR Centenarian*[TW]	Aged
Cancer	“Neoplasms”[MeSH] OR cancer[TW] OR neoplasm*[TW] OR neoplasia*[TW] OR tumour*[TW] OR tumor*[TW] OR malignan*[TW] OR carcinom*[TW] OR adenocarcinom*[TW] OR sarcom*[TW] OR leukemi*[TW] OR leukaemi*[TW] OR lymphoma*[TW] OR *blastoma*[TW] OR melanoma*[TW]	Neoplasms

MeSHMedical Subject Headings

### Study selection

The software Rayyan will be used in the screening process. The selection of studies will follow a three-stage process:

1. Screening by title and abstract: duplicate records will be removed before initial screening. Independent reviews will be conducted in reviewer pairs to screen the references. Disagreements will be resolved by a third reviewer.

2. Screening by full text: following the title and abstract screening, the full texts of the retained articles will be retrieved and independently screened by a reviewer pair based on the eligibility criteria. Disagreements will be resolved through the same process as the title and abstract screening.

3. Screening by references: the reference list of all included articles will be screened by a reviewer.

### Data management (included and excluded papers)

All excluded articles during full-text screening will be listed on a digital spreadsheet with the reason for exclusion. EndNote V.21 will be used for reference management and bibliography generation.

If a potentially relevant study is only available as an abstract, we will contact the author of the study to ask whether the full version of the manuscript has been published elsewhere; if not, the study will be excluded. We limit contact with the authors to twice within 2 weeks. If the authors do not respond within 2 weeks of the second email, the study will be excluded.

### Data extraction

For each included paper, two reviewers will independently extract at least the following data:

- Author(s)
- Year of publication
- Objectives of the study
- Study design (eg, quantitative study, qualitative study, mixed method, intervention study, cross-sectional study)
- Country of the study
- Sampling method (eg, random, convenience sampling, snowballing) if appropriate.
- Sampling size
- Characteristics of the sample (eg, age, sex/gender, cancer type(s), study setting (community, nursing home and hospital))
- Description of Online Social Support Intervention
  - Platform used
  - Features provided (eg, chat rooms, community forums, links to resources etc)
  - Description of comparative groups (eg, by age or intervention group, if applicable)
- Outcome measures (eg, changes in social support, psychological outcomes, quality of life and feasibility factors such as the acceptability of online social support)
  - Instrument measurement (eg, questionnaire, survey)

- Key findings

During the data extraction process, if there are any disagreements with the data items, the same review process used for the screening procedures will be conducted.

The extracted data will be tabulated and summarised in a narrative summary following the ‘descriptive-analytical’ method.^27^ This stipulates the application of a common framework to all included papers to collect standard information on the key components and findings. The findings are then presented in a structured narrative format. The results will provide an overview of online social support for older adults with cancer.

### Quality assessment

No quality assessment will be performed, as suggested by Joanna Briggs Institute and the Arksey and O’Malley Framework.^27 30^ Two reviewers will execute the entire process independently. The third reviewer will resolve any conflicts thereby mitigating potential biases in inclusion where possible.

### Implications

The findings of this scoping review will provide an understanding of the current state of online social support for older adults with cancer, identify gaps to improve interventions and provide valuable information about online social support for them. Future research building on this review’s findings could lead to the development of tailored digital tools for social support. These tools may provide clinicians with additional resources to recommend as a form of protective social prescription for older adults with cancer.

## Ethics and dissemination

This review does not require ethical approval as it involves the analysis of secondary data. The results of this review will be published in a peer-reviewed journal and presented at conferences.
