# Supplementary material for: Exploring the status of online social support for older adults with cancer: a scoping review protocol
Source: BMJ Open. 2024 Dec 26;14(12):e087251. doi: 10.1136/bmjopen-2024-087251 (PMC11683909; doi:10.1136/bmjopen-2024-087251)
Supplement: Supplementary file 1 [file bmjopen-2024-087251.reviewer_comments.pdf]

PEER REVIEW HISTORY

BMJ Open publishes all reviews undertaken for accepted manuscripts. Reviewers are asked to complete a checklist review form and are provided with free text boxes to elaborate on their assessment. These free text comments are reproduced below.

ARTICLE DETAILS

Title (Provisional)

Exploring the status of online social support for older adults with cancer: A Scoping Review Protocol

Authors

Liu, Fei; Pilleron, Sophie; Pinker, India

VERSION 1 - REVIEW

**Reviewer**	**1**
**Name**	**Suarilah, Ira**
**Affiliation**	**Universitas Airlangga**
**Date**	**03-May-2024**
**COI**	**None to declare**

Dear Author(s),

It is an interesting scooping review protocol exploring the status of online social support for older adults with cancer: A Scoping Review Protocol.

Overall, everything is well written. However, more limitations are reported.
Hereby, I explain my concerns in detail as written below.

Abstract:
Introduction:
Page 2, line 30-31: The sentence is arguing. What is the most logical reason for increasing number of older adults with cancer have access to social support through online technologies; Due to COVID-19 or the advancement of information and technology?
Page 2, line 32:
Why social support? Author should develop a critical thinking to explain the urgency of exploring this particular factor in older adults with cancer.
Page 3, line 36: I suggest author to add one database namely PysInfo to gain more relevant studies.
Page 3, Lines 49-50: Concerning no language limitation was made, authors should consider the project is supported by language(s) experts.

I suggest author(s) to revise the whole body of the manuscript first, later rewrite the abstract.

Introduction
Page 4, line 60-65:
Please, add the strengths of social support through online technologies in older adults with cancer which cannot find in conventional face to face?

Method and Analysis
Concerning no language limitation was made, Authors should consider the project is supported by language(s) experts.
Do the authors focused on general cancer or diseases/illness associated cancer?

Page 7, lines 7-15
Do authors include older adults with dementia? also older adults with psychiatric disorder?
I would Author(s) to revise the inclusion and exclusion criteria/PICO's framework

Page 8, line 124-125
Please add one database namely PysInfo to gain more relevant studies.

Data extraction
Page 10, line 166: I suggest author to add study setting (community, nursing home, hospital)
Page 10, line 173: What is acceptability as written here for outcomes measure? Is it about technology acceptance?
Page 10, line 174: I would like Author to consider the instruments to measure online social support applied in the included studies.

**Reviewer**	**2**
**Name**	**Liao, Kuang-Ming**
**Affiliation**	**Chi Mei Hospital Chiali**
**Date**	**11-May-2024**
**COI**	**No competing interests.**

As this is a scoping review protocol, it is designed to outline the methodology for exploring the status of online social support for older adults with cancer. The protocol specifies how the data will be collected, screened, and analyzed. It provides a comprehensive plan, but actual conclusions will only be drawn once the review is conducted.

The authors address possible limitations of the research. They acknowledge that one limitation of the scoping review is the exclusion of grey literature from the search strategy. This could potentially omit relevant studies that are not published in peer-reviewed journals.

The accuracy of data collection and interpretation will be assessed based on the actual review process described in the protocol. The authors have outlined a thorough method for data extraction and screening, involving multiple reviewers to ensure accuracy and resolve conflicts.

The protocol is structured according to the Preferred Reporting Items for Systematic Reviews and Meta-Analyses extension for Scoping Reviews guidelines. The use of these guidelines ensures that the protocol is comprehensive and transparent.

The authors have provided detailed information on the eligibility criteria, search strategy, study selection process, and data extraction methods . They also mention the use of specific databases and a health-focused research librarian to ensure the search strategy is comprehensive. These details provide sufficient information to validate the study and ensure its reproducible.

Provide a more detailed discussion on how potential biases will be addressed during the review process. This includes selection bias, publication bias, and any biases introduced by the exclusion of non-English studies (if applicable).

Expand the strengths and limitations section to provide a balanced view of the protocol. While strengths are mentioned, a more detailed exploration of the limitations and how they will be mitigated would add depth to the protocol.

**Reviewer**	**3**
**Name**	**Kaur, Anahat**
**Affiliation**	**Albert Einstein College of Medicine**
**Date**	**27-Aug-2024**
**COI**	**No competing interests to declare**

The protocol is well-structured and provides a solid foundation for the scoping review. Addressing the suggested clarifications could strengthen the protocol and enhance the quality and impact of the review.

Methods and Analysis:

-The description of the search strategy is adequate, but it would be helpful to specify if any effort will be made to update the search close to the final synthesis of the review.

-The narrative synthesis approach is appropriate, but consider providing more detail on how themes or patterns will be identified and analyzed. Will qualitative analysis methods (e.g., thematic analysis) be employed?

-While the decision not to perform a quality assessment is justified by the methodology, it would be helpful to acknowledge the potential limitations this might introduce in interpreting the findings.

Ethics and dissemination:

-This section could benefit from a statement on how the findings will be used beyond publication and conferences, such as influencing policy or practice.

Strengths and Limitations:

- This section mentions that the study is the first of its kind and that grey literature is excluded. However, further elaboration on how these factors might affect the generalizability of the findings would be valuable.

- Consider adding a point on how the findings might inform future research or clinical practice.

VERSION 1 - AUTHOR RESPONSE

Reviewer: 1
Dr. Ira Suarilah, Universitas Airlangga
Comments to the Author:
"Dear Author(s),
It is an interesting scooping review protocol exploring the status of online social support for older adults with cancer: A Scoping Review Protocol. Overall, everything is well written. However, more limitations are reported. Hereby, I explain my concerns in detail as attached. (See attached file BMJ Open-Review May 2024.pdf (26 K) (Author and Editor))"

"Abstract: Introduction: Page 2, line 30-31: The sentence is arguing. What is the most logical reason for increasing number of older adults with cancer have access to social support through online technologies; Due to COVID-19 or the advancement of information and Technology?"

Our response: Thank you for your review and suggestions. We have modified it to enhance the clarity and address both this point as well as your following comment in Line 32 (please see below).

"Page 2, line 32: Why social support? Author should develop a critical thinking to explain the urgency of exploring this particular factor in older adults with cancer."
Our response: Thank you for your suggestion. We agree that it should be elucidated. We revised it based on the two above comments:
" Notably, an increasing number of older adults with cancer have access to social support through online technologies, especially since the COVID-19 pandemic, which has accelerated the need for online social support." —Line 30-32

"Page 3, line 36: I suggest author to add one database namely PysInfo to gain more relevant studies."
Our response: Thank you for your constructive suggestion. We expand on this in response to your comment below. We are primarily limited by the databases to which

our institution is subscribed. Our databases were selected by our dedicated and expert research librarian who suggested these data based for optimum literature coverage - including studies that would be held under the PsycInfo database.

"Page 3, Lines 49-50: Concerning no language limitation was made, authors should consider the project is supported by language(s) experts."
Our response: Thank you for highlighting this. We have added further justification as follows:
" * Authors' institution hosts diverse language expertise to support this criteria"---Line 134

"I suggest author(s) to revise the whole body of the manuscript first, later rewrite the abstract."
Our response: Thank you for your suggestion. We have revised the manuscript following your insights.

"Introduction, Page 4, line 60-65: Please, add the strengths of social support through online technologies in older adults with cancer which cannot find in conventional face to face?"
Our response: Thank you for your suggestion. We have further justified the strengths of online social support through the following:
"In addition to face-to-face support that addresses their complex needs, online social support can also provide a sense of connection and community, regardless of geographic constraints (30). As such, vulnerable and frail older adults with cancer, in particular those who may not be able to travel easily, may benefit from social support provided through these online technologies"---Line 70-75

"Method and Analysis, Concerning no language limitation was made, Authors should consider the project is supported by language(s) experts."
Our response: Thank you for your constructive suggestion. The work was conducted at an institute hosting researchers with over 50 nationalities. As such, we had adequate linguistic support. We have highlighted this now, as further justification to our PICO framework-based inclusion criteria:
" * Authors' institution hosts diverse language expertise to support this criteria"

"Do the authors focused on general cancer or diseases/illness associated cancer?"
Our response: Thank you for the comment. We focused on general cancer only. The presence of other diseases (associated or otherwise), however, was not an exclusion criteria. The primary basis of inclusion was being diagnosed with cancer. We recognise, however, that we have not made this clear and have duly added further clarity to our inclusion criteria under the subsection "*Participants*": "No restriction on co-morbidities."

"Page 7, lines 7-15; Do authors include older adults with dementia? also older adults with psychiatric Disorder?"
Our response: Thank you for your insightful question. The primary inclusion criterion for participants in the studies was a cancer diagnosis in older adults, while the presence of other comorbid conditions did not result in exclusion. As such, no restriction was placed on cognitive ability.

"I would Author(s) to revise the inclusion and exclusion criteria/PICO's framework"
Our response: Thank you for your suggestion. We have updated the relevant points according to your valuable insights.

"Page 8, line 124-125; Please add one database namely PysInfo to gain more relevant studies."
Our response: Thank you for your suggestion. Our research librarian with database expertise assisted with the selection of these databases for optimum relevant study coverage. We are primarily limited by the databases to which our institution is subscribed, which does not include PsycInfo, unfortunately. We had already raised PsycInfo with our librarian and she suggested that the relevant studies from PsycInfo are covered by EBSCO CINAHL and Embase as their data coverage significantly overlaps, this is further corroborated by research that suggests EBSCO CINAHL significantly overlaps with PsycInfo as they have the same host (Hirt et al., 2021). Furthermore, as we explained in our cover letter, the work was time-restricted and now completed, further limiting our ability to add this database at this time.
We have, however, clarified the database selection to be as a result of the advice of our research librarian in the manuscript to highlight this. Though we recognise this is a limitation of the review.
"We will search for articles from PubMed, Elsevier Embase (including Medline), and EBSCO CINAHL Complete, as advised by our in-house research librarian for optimum literature coverage across the institutionally-available databases."---Line 137-138

"Data extraction- Page 10, line 166: I suggest author to add study setting (community, nursing home, hospital)"
Our response: Thank you for your suggestion. This was intended for inclusion via "Characteristics of the sample" but we recognise this is limited in clarity. We have since added this to our "*Data extraction*" subsection.

"Page 10, line 173: What is acceptability as written here for outcomes measure? Is it about technology acceptance?"
Our response: Thank you for your insights. The acceptability was referring to online social support tools, we have since added this description for further clarification.

"Page 10, line 174: I would like Author to consider the instruments to measure online social support applied in the included studies."

Our response: Thank you for your diligent insights. We intended to include this information in "outcome measures" but we recognise this was not clear enough. We have now shown this consideration by adding this specificity to our "*data extraction*" subsection.

Reviewer: 2
Dr. Kuang-Ming Liao, Chi Mei Hospital Chiali
Comments to the Author:
"As this is a scoping review protocol, it is designed to outline the methodology for exploring the status of online social support for older adults with cancer. The protocol specifies how the data will be collected, screened, and analyzed. It provides a comprehensive plan, but actual conclusions will only be drawn once the review is conducted."
Our response: Thank you for taking the time to review this protocol and for your insightful suggestions.

"The authors address possible limitations of the research. They acknowledge that one limitation of the scoping review is the exclusion of grey literature from the search strategy. This could potentially omit relevant studies that are not published in peer-reviewed journals."
Our response: Thank you for highlighting this. We agree and have highlighted this more clearly in the limitations section.

"The accuracy of data collection and interpretation will be assessed based on the actual review process described in the protocol. The authors have outlined a thorough method for data extraction and screening, involving multiple reviewers to ensure accuracy and resolve conflicts. The protocol is structured according to the Preferred Reporting Items for Systematic Reviews and Meta-Analyses extension for Scoping Reviews guidelines. The use of these guidelines ensures that the protocol is comprehensive and transparent.
The authors have provided detailed information on the eligibility criteria, search strategy, study selection process, and data extraction methods . They also mention the use of specific databases and a health-focused research librarian to ensure the search strategy is comprehensive. These details provide sufficient information to validate the study and ensure its reproducible."
Our response: Thank you so much for taking the time to review our paper and for your positive feedback.

"Provide a more detailed discussion on how potential biases will be addressed during the review process. This includes selection bias, publication bias, and any biases introduced by the exclusion of non-English studies (if applicable)."
Our response: Thank you for your suggestion. We have further expanded our quality assessment subsection to reflect how our search strategy could mitigate selection bias. No languages were excluded for this review as our institution has sufficient linguistic support for this. We have outlined this now in relation to our

inclusion criteria. As this scoping review does not include grey literature, there may be publication bias but this has since been highlighted as a limitation. In addition, in the write-up of the full article, biases were further explored in the discussion and limitations section.

"Expand the strengths and limitations section to provide a balanced view of the protocol. While strengths are mentioned, a more detailed exploration of the limitations and how they will be mitigated would add depth to the protocol."

Our response: Thank you for your suggestion. We have expanded our limitation section as follows:

"The lack of a quality assessment, as stipulated by the chosen methodology, may introduce biases due to variations in the quality of the included articles, potentially limiting the generalisability of the findings."

Reviewer: 3

Dr. Anahat Kaur, Albert Einstein College of Medicine

Comments to the Author:

"The protocol is well-structured and provides a solid foundation for the scoping review. Addressing the suggested clarifications could strengthen the protocol and enhance the quality and impact of the review."

Our response: Thank you so much for your time and thorough review.

"Methods and Analysis:

The description of the search strategy is adequate, but it would be helpful to specify if any effort will be made to update the search close to the final synthesis of the review."

Our response: Thank you for your suggestion. We did run a second, updated search prior to analysis. We have since specified this in the manuscript:

"To ensure the most up-to-date literature is included, this search will be run once more before final analysis to ensure we capture as much relevant literature as possible"

"The narrative synthesis approach is appropriate, but consider providing more detail on how themes or patterns will be identified and analyzed. Will qualitative analysis methods (e.g., thematic analysis) be employed?"

Our response: Thank you for your question. In accordance with the methodology outlined by Arksey and O'Malley, we used the narrative synthesis methodology that we have since outlined in more detail:

"The extracted data will be tabulated and summarised in a narrative summary following the "descriptive-analytical" method within the narrative tradition (27). This stipulates the application of a common framework to all included papers to collect standard information on the key components and findings. The findings are then presented in a structured narrative format." - Line 197 -201

“While the decision not to perform a quality assessment is justified by the methodology, it would be helpful to acknowledge the potential limitations this might introduce in interpreting the findings.”

Our response: Thank you for highlighting this. We agree and have expanded the limitations section accordingly:
“The lack of a quality assessment in the methodology may introduce biases due to variations in the quality of the included articles, potentially limiting the generalisability of the findings.”

“Ethics and dissemination:
This section could benefit from a statement on how the findings will be used beyond publication and conferences, such as influencing policy or practice.”

Our response: Thank you for your suggestion. We agree and have added the following to the subsection “*Implications*”:
“Future research building on this review's findings could lead to the development of tailored digital tools for social support. These tools may provide clinicians with additional resources to recommend as a form of protective social prescription for older adults with cancer.”

“Strengths and Limitations:
This section mentions that the study is the first of its kind and that grey literature is excluded. However, further elaboration on how these factors might affect the generalizability of the findings would be valuable.”

Our response: Thank you for your suggestion. We have added this insight to our limitations.

“Consider adding a point on how the findings might inform future research or clinical practice.”

Our response: Thank you for your suggestion. We agree and we have incorporated this into our response to your previous comment on implications.

VERSION 2 - REVIEW

**Reviewer**	**1**
**Name**	**Suarilah, Ira**
**Affiliation**	**Universitas Airlangga**
**Date**	**28-Nov-2024**
**COI**	

I appreciate the revisions made. The necessary adjustments is strengthen your manuscript.

**Reviewer**	**3**
**Name**	**Kaur, Anahat**
**Affiliation**	**Albert Einstein College of Medicine**
**Date**	**02-Dec-2024**
**COI**	

Authors have addressed all concerns raised during initial review. No further comments. I look forward to future publications with results from this study.
